# BIOPSYCHOSOCIAL DETERMINANTS OF NEUROCOGNITIVE IMPAIRMENT IN THE ENGLISH LONGITUDINAL STUDY OF AGEING

**DOI:** 10.1093/geroni/igz038.683

**Published:** 2019-11-08

**Authors:** Dorina Cadar, Dorina Cadar, Jessica Abell, David J Llewellyn, Andrew Steptoe

**Affiliations:** 1 University College London, London, United Kingdom; 2 University College London,, London, England, United Kingdom; 3 University of Exeter Medical School, Exeter, England, United Kingdom; 4 University College London, london, England, United Kingdom

## Abstract

Biological and psychosocial risk factors, particularly those that are malleable across the life course, are important determinants of neurocognitive health in later life. We investigated several determinants of cognitive impairment using the Mini-Mental Status Examination (MMSE), as part of the Harmonised Cognitive Assessment Protocol in 1,200 individuals aged ≥65 years from the English Longitudinal Study of Ageing. More than half the participants (55%) were married, 15% had diabetes, 12% had CHD, and fewer than 10% had a stroke. A longitudinal investigation of various risk factors measured at wave 6 (2012-13) was conducted in relation to neurocognitive impairment ascertained with the MMSE ≤24 in 2018. Our results indicate that certain environmental compensatory factors such as education, a marker of cognitive reserve, wealth and psychological wellbeing are relevant determinants of subsequent neurocognitive impairment six years later. These findings are highly informative for the development of interventions aiming to maintain neurocognitive health.

